# Understanding psychotic-like experiences in children in the context of dimensions of psychological problems

**DOI:** 10.3389/frcha.2024.1410804

**Published:** 2024-08-02

**Authors:** Hee Jung Jeong, Benjamin B. Lahey, Gabrielle E. Reimann, E. Leighton Durham, Camille Archer, Tyler M. Moore, Krisha Shah, Antonia N. Kaczkurkin

**Affiliations:** ^1^ Department of Psychology, Vanderbilt University, Nashville, TN, United States; ^2^ Department of Health Studies, University of Chicago, Chicago, IL, United States; ^3^ Department of Psychiatry and Behavioral Neuroscience, University of Chicago, Chicago, IL, United States; ^4^ Department of Psychiatry, Perelman School of Medicine, University of Pennsylvania, Philadelphia, PA, United States

**Keywords:** psychotic-like experience, general psychopathology, ADHD, conduct problems, children

## Abstract

**Introduction:**

Although psychotic behaviors can be difficult to assess in children, early identification of children at high risk for the emergence of psychotic symptoms may facilitate the prevention of related disorders. Psychotic-like experiences (PLEs), or subthreshold thought and perceptual disturbances, could be early manifestations of psychosis that may predict a future diagnosis of a psychosis-related disorder or nonspecific correlates of a wide range of psychological problems. Additional research is needed regarding how PLEs map onto dimensions of psychopathology in children.

**Methods:**

In the present study, we examined the association between PLEs and general and specific dimensions of psychological problems in a sample of 10,692 children from the Adolescent Brain Cognitive Development Study (ABCD Study).

**Results:**

The results of this study showed that self-reported PLEs were associated with a general psychopathology factor and an ADHD factor, which were defined in hierarchical models of parent-rated psychological problems.

**Discussion:**

These findings suggest that PLEs are broadly associated with a wide range of psychological problems through the general psychopathology factor even before psychotic disorders typically manifest. This study supports the need for longitudinal analyses of future waves of the ABCD Study to determine if PLEs can detect children at high risk for serious psychological problems in adulthood.

## Introduction

Psychotic behaviors that meet diagnostic criteria for schizophrenia or other mental health disorders are difficult to assess in children as the assessment requires specialized skills and it may be difficult to fully capture these symptoms in children with a relatively limited ability to share their experiences. Yet there is a sharp increase in the incidence of diagnosed psychotic disorders beginning in late adolescence (1), suggesting that detection of children at high risk for the emergence of serious psychotic behavior before the manifestation of a diagnosis could facilitate early identification and prevention efforts. One possible risk factor for the onset of psychosis in the future is self-reported psychotic-like experiences (PLEs) during childhood. PLEs refer to psychotic-related symptoms reported in non-clinical samples (2), with a prevalence estimated to be 5%–8% in the general population (3). The concept of PLEs has received attention due to an increased recognition that psychotic experiences exist on a continuum from few symptoms to many symptoms, suggesting the existence of a spectrum of psychosis symptoms (4). The prevalence of PLEs is estimated to be higher in children than in the general population, with increased rates in younger children than older children (5). Specifically, some children may report hearing strange sounds or having unusual sensations, being distrustful of others, believing that they have magical powers or that their mind is trying to trick them, or other atypical perceptions that resemble psychotic-related experiences (6–9). The meaning of these experiences is unclear, especially as most of these symptoms disappear over time in children (10), suggesting that some PLEs are not necessarily associated with any specific risk for psychopathology. However, there are two possibilities regarding how PLEs in children may be related to psychopathology.

First, PLEs could be early manifestations of psychosis that may predict a future diagnosis of a psychotic disorder in a subset of children who experience these perceptions (3, 6, 11, 12). Second, there is emerging evidence that PLEs may be nonspecific presentations of a wide range of internalizing and externalizing psychological problems (13–15). Furthermore, the broad range of dimensions of psychological problems that are associated with PLEs raises the possibility that they are associated with a general factor (or p factor) of psychological problems (16–18). Several researchers have proposed hierarchical models of the structure of correlated dimensions of psychological problems (8, 16, 19, 20). Although these models differ in some important ways, including the specific statistical approaches used to describe the correlational structure, each model assumes that the shared variance across all symptoms is captured by a general factor of psychopathology.

These two explanations for the existence of PLEs (that PLEs index an early manifestation of psychosis or that PLEs are nonspecifically associated with general psychopathology) are not necessarily mutually exclusive. PLEs could indicate early signs of a future diagnosis of psychosis and be linked to general mental health issues at the same time. This is supported by prior work suggesting that severe psychotic experiences are associated with overall mental health problems. In particular, problematic psychotic experiences considered to be symptoms of schizophrenia, schizotypal personality disorder, and mania such as the individual being “out of touch with reality” have been proposed to be strongly associated with the general factor of psychopathology (20). Likewise, Caspi and Moffitt proposed that disordered thought (illogical and distorted thinking) is one of the dysfunctional processes underlying the general factor of psychological problems (17). Thus, PLEs might indicate both early symptoms of psychosis and a component of a general psychopathology factor. While it should be noted that some PLEs reported in children are normative and may not be associated with mental health diagnoses, it is possible that PLEs can reflect both early psychosis symptoms and general mental health problems.

These hypotheses are tentatively supported by previous findings from several studies of adolescents and adults showing that symptoms of psychosis load robustly onto the general factor of psychopathology (16, 20–22). Furthermore, there is growing evidence from family and molecular studies that schizophrenia shares much of its genetic risk with a broad range of other mental health disorders (23–25) and with PLEs (26). This suggests that at least some of the genetic and environmental variance that gives rise to disordered thought is shared with essentially all other dimensions of psychological problems, as represented by a general psychopathology factor. However, this work has been primarily conducted in adolescents and adults; it remains unclear whether these associations are apparent in children.

Additionally, prior work suggesting that psychosis is associated with a general factor of psychopathology was limited because it only examined the association between psychotic behavior and the general factor, neglecting PLEs and specific factors such as internalizing and externalizing dimensions (16, 27). When examining associations between PLEs and the general or specific factors, different interpretations may arise depending on the hierarchical model used (28). In a bifactor model, every item loads on both the general factor and one specific factor, thereby partitioning the total explained variance in each item between the general and specific factors (29–31). Thus, dimensions like conduct problems and attention deficit/hyperactivity disorder (ADHD) are orthogonal (uncorrelated) when defined in a bifactor model because the shared variance among all items is allocated to the general factor (30–32). In contrast, in a higher-order model, each item loads on one of several correlated lower-order factors and the lower-order factors then load onto a higher-order general factor (19). In this model, the general factor is defined by the variance shared by the lower-order factors, and there is no direct relationship between items and the general factor.

Despite the growing number of studies using dimensional models to define psychopathology, there has been a dearth of work examining the relationship between PLEs and psychopathology dimensions in children. Thus, the purpose of the current study was to examine the association between PLEs and dimensions of psychopathology measured with bifactor and higher-order models in a large sample of children. Since the meaning of the association between the general and specific factors and PLEs changes based on the hierarchical modeling being used, the current study advances our understanding of PLEs in children by examining their association with the general and specific dimensions of psychological problems defined using two different models. Based on previous findings, it was hypothesized that PLEs would be associated with the general psychopathology factor. The associations between PLEs and the specific factors were exploratory.

## Methods

### Participants

The present analyses used data from 10,692 participants (48.02% female) from wave 1 (collected from September 2016 to June 2018) of the Adolescent Brain Cognitive Development^SM^ Study (ABCD Study®; release 4.0). Participants with missing data on parent ratings of psychological problems, age, sex, race-ethnicity, and non-participation and post-stratification weight variables were excluded from analyses. The sample was recruited at 22 sites across the United States at 9–10 years of age as part of a planned longitudinal study. The sites do not perfectly represent the population of the United States, but the same unbiased recruitment process was used within every site (33). Furthermore, the ABCD Study provides post-stratification weights which are used to adjust the sample to better approximate population parameters in terms of demographics such as sex, race/ethnicity, and family income (34). These post-stratification weights were applied in all analyses. Most (66.42%) participants were one child of singleton birth from different families, but some had a twin or non-twin sibling in the study, so family was taken into account (see Data Analyses section). Parents classified their children as non-Hispanic White (52.5%), Hispanic (20.53%), Black (14.5%), and other racial-ethnic groups (12.48%). See Table 1 for additional details about the demographic breakdown of the sample.

**Table 1:** Summary of the demographic characteristics of the sample (*N* = 10,692).

	Mean	SD
Age (months)	119.07	7.50
	*N*	*%*
Gender		
Female	5,134	48.02
Male	5,558	51.98
Race/Ethnicity		
White	5,613	52.50
Hispanic	2,195	20.53
Black	1,550	14.50
Other	1,334	12.48
Sibling status		
Single birth	7,102	66.42
Sibling	1,640	15.34
Twin	1,922	17.98
Triplet	28	0.26
Household annual income		
<$5,000	354	3.31
$5,000–$11,999	376	3.52
$12,000–$15,999	254	2.38
$16,000–$24,999	465	4.35
$25,000–$34,999	584	5.46
$35,000–$49,999	826	7.73
$50,000–$74,999	1,350	12.63
$75,000–$99,999	1,444	13.51
$100,000–$199,999	3,006	28.11
>$200,000	1,133	10.60
Missing	900	8.42
Parental education		
No degree	540	5.05
High school degree/GED	1,284	12.01
Some college	1,750	16.37
Associate's degree	1,385	12.95
Bachelor's degree	3,039	28.42
Master's degree	2,055	18.75
Professional/Doctoral degree	639	5.98

The “Other” Race/Ethnicity category includes those who were identified by their parent as American Indian/Native American, Alaska Native, Native Hawaiian, Guamanian, Samoan, Other Pacific Islander, Asian Indian, Chinese, Filipino, Japanese, Korean, Vietnamese, Other Asian, or Other Race.

### Measures

The Child Behavior Checklist (CBCL) (35) is a parent rating scale of child behavior consisting of 119 symptoms (113 items with some items representing multiple symptoms) describing problem behaviors and emotions on a scale of 0 = not true (as far as you know), 1 = somewhat or sometimes true, or 2 = very true or often true. Missing data on CBCL items was <0.1%. PLEs were measured using the Prodromal Questionnaire–Brief Child Version (PQ-BC), which has been shown to be a valid measure of PLEs in children (7). Children reported whether they had experienced each of 21 psychotic-like experiences and then rated the extent to which the endorsed experience bothered them on a 1–5 scale. The PQ-BC has been shown to have adequate internal consistency, exhibit measurement invariance across the sexes and racial/ethnic groups, and exhibit construct validity in a subset of the ABCD Study sample (7). Furthermore, the PQ-BC has been shown to be associated with a family history of psychotic disorder specifically, but not depression or mania (7). This measure is also associated with other known correlates of PLEs including internalizing and externalizing symptoms, neuropsychological test performance deficits, and motor and speech developmental delays (7). For the current analysis, the total number of PLEs, which is the sum of endorsed questions (0 = No; 1 = Yes; possible range = 0–21) and distress-weighted PLEs, which is the total number of endorsed questions (0 = No; 1 = Yes) weighted by level of distress (1 = Not very bothered; 2 = Slightly bothered; 3 = Moderately bothered; 4 = Very much bothered; 5 = Extremely bothered; possible range = 0–126) were used.

### Data analyses

**Figure 1:**
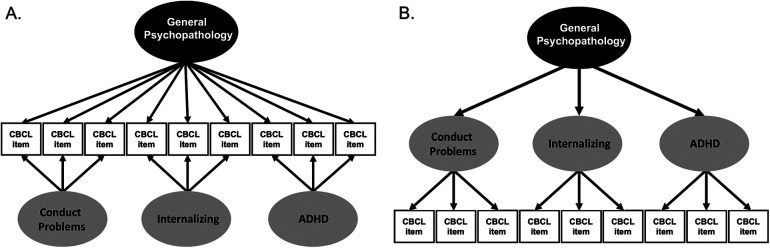
A diagram of the bifactor and higher-order models. (**A**) In a bifactor model, each CBCL item loads both onto a general factor and only one of the specific factors. All factors are orthogonal to each other. (**B**) In a higher-order model, each CBCL item loads onto lower-order factors, which then load onto a general factor. The lower-order factors in the higher-order model are allowed to correlate.

Structural equation modeling (SEM) was conducted in Mplus 8.4 using the mean- and variance-adjusted weighted least squares (WSLMV) estimator and pairwise deletion for missing data (36). All analyses included clustering based on family membership to account for siblings and multiple births in the sample, stratification by site to account for data collection across 21 sites, and weighting by post-stratification weights to make the sample more representative of the U.S. population. In our previous study (37), exploratory structural equation modeling identified three psychopathology dimensions: conduct problems, internalizing symptoms, and ADHD symptoms. We examined the hierarchical structure of the CBCL items using two models: a bifactor model and a higher-order model. In a bifactor model (Figure 1A), every CBCL item loads onto both the general factor and on one and only one orthogonal specific factor obtained from exploratory analyses. In a bifactor model, the total explained variance in each item is partitioned between the general and specific factors (29–31) so that the general factor and specific factors are uncorrelated with one another. In a higher-order model (Figure 1B), each item loads onto one of several correlated lower-order factors and the lower-order factors load onto a higher-order general factor. The general and specific factors in the bifactor and the higher-order models have been shown to exhibit adequate construct reliability and estimated replicability and demonstrate robust criterion validity in a random half of the ABCD Study sample (37).

SEM was performed to examine the association between PLEs and the general and specific factors of psychopathology. The total number of PLEs and distress-weighted PLEs were predicted by the general and specific factors of psychopathology controlling for demographic covariates (age, sex, and race/ethnicity). Using a bifactor model, the general factor and specific factors were included in the model at the same time, which is possible because the factors are orthogonal. Due to the perfect collinearity between the general factor and the specific factors defined through higher-order modeling, separate analyses were performed with the general factor and the specific factors predicting PLEs. Sensitivity analyses were performed controlling for parental education and family income to account for socioeconomic status. To correct for multiple comparisons, the false discovery rate (q < 0.05) was used to the adjust *p*-values across each factor obtained through bifactor and higher-order models. The analysis code is available at https://github.com/VU-BRAINS-lab/ABCD-PLE.

## Results

The general and specific factors defined in a bifactor model exhibited adequate determinacy, construct replicability, and other psychometric properties, as did the general and lower-order factors defined in a higher-order model (Table 2). Using a bifactor model that controlled for age, sex, and race/ethnicity and after correction for multiple comparisons, the results showed that PLEs were significantly associated with the general psychopathology, conduct problems, and ADHD factors (Table 3). When parental education and family income were added as covariates, the magnitudes of the associations with the general and ADHD factors decreased but were still statistically significant (Table 3). In contrast, the specific conduct problems factor was no longer significantly associated with PLEs after controlling for socioeconomic variables. The same pattern of significant findings was found when children's self-reported PLEs were weighted by ratings of how much the experiences upset the child—the general and ADHD factors remained significant but the specific conduct problems was not associated with distress-weighted PLEs (Table 3).

**Table 2:** Psychometric indices for each factor in the bifactor and the higher-order models.

Index	General	Conduct problems	Internalizing	ADHD
Bifactor model				
H	0.977	0.853	0.852	0.732
ECV (S&E)	0.695	0.129	0.116	0.060
ECV (New)	0.695	0.251	0.465	0.251
Omega	0.982	0.974	0.936	0.946
OmegaH	0.864	0.203	0.437	0.216
Factor determinacy	0.975	0.917	0.925	0.893
PUC	0.640	–	–	–
Higher-Order model				
H	–	0.974	0.947	0.951
Factor determinacy	0.947	0.988	0.974	0.977

H, index of construct replicability; ECV, explained common variance; PUC, percent correlations that are uncontaminated; OmegaH, *ω* hierarchical.

**Table 3:** Primary and sensitivity analyses testing associations of general and specific factors of psychological problems defined in either bifactor or higher-order models with two measures of psychotic-like experiences, adjusting for demographic covariates.

Primary analyses: adjusting for age, sex, and race-ethnicity (*N* = 10,692).						
Predictor	β	*S.E.*	*p_fdr_*	β	*S.E.*	*p_fdr_*
	Bifactor models					
	Number of PLEs			Distress-weighted PLEs		
General^a^	**0**.**126**	**0**.**014**	**0**.**000**	**0**.**133**	**0**.**014**	**0**.**000**
Conduct problems^a^	**0**.**041**	**0**.**017**	**0**.**021**	0.034	0.017	0.056
Internalizing^a^	−0.012	0.014	0.409	−0.007	0.014	0.635
ADHD^a^	**0**.**070**	**0**.**018**	**0**.**000**	**0**.**057**	**0**.**017**	**0**.**000**
	Higher-order models					
	Number of PLEs			Distress-weighted PLEs		
General^b^	**0**.**146**	**0**.**013**	**0**.**000**	**0**.**150**	**0**.**013**	**0**.**000**
Conduct problems^c^	0.036	0.024	0.181	0.041	0.023	0.107
Internalizing^c^	−0.018	0.021	0.375	−0.006	0.020	0.772
ADHD^c^	**0**.**131**	**0**.**024**	**0**.**000**	**0**.**118**	**0**.**023**	**0**.**000**
Sensitivity analyses: adjusting for age, sex, race-ethnicity, parental education, and family income (*N* = 9,792).						
	Bifactor models					
	Number of PLEs			Distress-weighted PLEs		
General^a^	**0**.**119**	**0**.**015**	**0**.**000**	**0**.**125**	**0**.**015**	**0**.**000**
Conduct Problems^a^	0.031	0.017	0.099	0.025	0.017	0.197
Internalizing^a^	−0.014	0.015	0.363	−0.006	0.015	0.672
ADHD^a^	**0**.**071**	**0**.**018**	**0**.**000**	**0**.**061**	**0**.**018**	**0**.**002**
	Higher-order Models					
	Number of PLEs			Distress-weighted PLEs		
General^b^	**0**.**137**	**0**.**014**	**0**.**000**	**0**.**141**	**0**.**013**	**0**.**000**
Conduct Problems^c^	0.018	0.024	0.512	0.020	0.024	0.523
Internalizing^c^	−0.014	0.021	0.512	−0.001	0.020	0.954
ADHD^c^	**0**.**137**	**0**.**024**	**0**.**000**	**0**.**127**	**0**.**024**	**0**.**000**

Coefficients in bold are significant after FDR correction across sums of psychotic-like experiences and distress-weight sums of psychotic-like experiences for each factor obtained through bifactor and higher-order models for primary analyses and sensitivity analyses.

^a^ Because the general and specific factors are orthogonal in bifactor models, each regression coefficient adjusts for all other factor scores.

^b^ Because of the complete collinearity of general with lower-order factors in higher-order models, the regression coefficient for the general factor does not reflect adjusting for the lower-order factor scores.

^c^ Because of the complete collinearity of general with lower-order factors in higher-order models, regression coefficients reflect adjusting for each other lower-order factor score, but do not reflect adjusting for the general factor score.

When PLEs were associated with the general factor defined in the higher-order model while accounting for age, sex, and race/ethnicity, both measures of PLEs (counts and distress-weighted) were significantly associated with the general factor (Table 3). When PLEs were related to the three lower-order factors defined in the higher-order model while accounting for the same covariates, both measures of PLEs (counts and distress-weighted) were significantly associated with the ADHD factor. The associations between measures of PLEs and the general factor and ADHD factor remained significant after controlling for parental education and family income (Table 3).

## Discussion

Using two different statistical models and controlling for socioeconomic-related covariates, the results of the current study showed that both the number of PLEs endorsed and the amount of distress associated with these PLEs were significantly associated with the general factor of psychological problems. This is consistent with previous observations that PLEs are associated with a broad range of mental health problems and provides support for the hypothesis that disordered thought may be associated with the general factor of psychological problems (16, 20). PLEs were also found to be associated with specific ADHD problems in both statistical models. An association with conduct problems was found, but this did not survive after accounting for socioeconomic status. Finally, no associations between PLEs and internalizing symptoms were found.

The relationship between PLEs the general factor of psychopathology suggests that the presence of PLEs in children could be an indicator of risk for overall psychopathology, regardless of whether psychosis is present or not. This is consistent with previous studies that found high rates of mood and anxiety disorders in a community sample who reported psychotic experiences (38, 39). In this regard, van Os and Reininghause (40) suggested that PLEs represent two constructs: (1) a specific phenotype of attenuated psychotic phenomena and (2) a trans-phenotype related to different domains of psychopathology such as positive, negative, affective, and disorganized domains. The current findings support the second construct defined by van Os and Reininghause by demonstrating an association between PLEs and the general factor of psychopathology in children. The future waves of the longitudinal ABCD Study will be helpful in identifying whether the PLEs continue to predict transdiagnostic psychopathological phenomena or whether a specific association with psychotic symptoms becomes apparent as these symptoms emerge in adolescence and young adulthood.

The current study also showed a consistent association between PLEs and ADHD symptoms. The association between PLEs and ADHD is interesting in light of previous evidence that ADHD in childhood is associated with an increased risk for later diagnoses of psychotic disorders (41–43). Furthermore, previous studies have found shared genetic (44) and environmental risk (45) between childhood ADHD and adult schizophrenia. To explain the link between childhood ADHD and adulthood psychosis, it has been suggested that motor, perceptual, and attentional difficulties that resemble ADHD symptoms in children are early manifestations of behavioral alterations related to schizophrenia (46). While the current study builds upon previous work by using a large sample of children and supports the specific association between ADHD and PLEs, PLEs are not the same as psychosis—additional evidence is needed to confirm the association between ADHD and the risk of psychosis using future time points in this sample.

The present findings are also notable because the significant associations between PLEs and dimensions of psychological problems were found across informants (i.e., parent-rated CBCL problems and youth-reported PLEs). This is important because it rules out common method variance as an explanation for the observed associations. Since parent and youth ratings of psychological problems only correlate moderately (47), perhaps because of differences in maturity, perspectives, and opportunities to observe the child's behavior, it will be important to examine these associations in later waves of the ABCD Study when measures of youth-reported psychological problems are available.

### Limitations

The current study has limitations that should be taken into consideration. First, although the measure used in the current study has been shown to be a valid measure of PLEs in children (7), it was originally developed to measure prodromal states (48) which are distinctive from PLEs. Thus, it would be helpful to replicate these findings using a dedicated measure of PLEs. Secondly, although PLEs represent a heterogeneous construct, we used a single summary score of PLEs which limits our ability to draw inferences regarding whether specific types of PLEs are related to a greater extent to each psychopathology dimension. Rather than relying on a total score, future work that delineates distinct dimensions of PLEs utilizing factor analysis may be useful for examining more nuanced associations with psychopathology in children.

## Conclusion

In conclusion, our findings on the association of PLEs with dimensions of psychopathology in children are consistent with the hypothesis that psychotic-like experiences share some variance with general psychological problems and ADHD symptoms. However, given that many children outgrown their reported PLEs by adolescence and adulthood, future work is needed to determine whether PLEs observed in childhood are associated with future psychotic diagnoses. The additional waves of the longitudinal ABCD Study will provide a unique opportunity to examine whether PLEs represent risk factors for psychotic behavior prospectively from childhood through young adulthood.

## Data Availability

Publicly available datasets were analyzed in this study. This data can be found here: https://nda.nih.gov/abcd.
